# The purification and characterization of ATP synthase complexes from the mitochondria of four fungal species

**DOI:** 10.1042/BJ20150197

**Published:** 2015-05-05

**Authors:** Sidong Liu, Thomas J. Charlesworth, John V. Bason, Martin G. Montgomery, Michael E. Harbour, Ian M. Fearnley, John E. Walker

**Affiliations:** *The Medical Research Council Mitochondrial Biology Unit, Cambridge Biomedical Campus, Hills Road, Cambridge CB2 0XY, U.K.

**Keywords:** ATP synthase, fungi, mitochondria, subunit composition, supernumerary subunits, thermal stability, 6.8PL, 6.8 kDa proteolipid, ACMA, amino-6-chloro-2-methoxyacridine, C_12_E_8_, octa(ethylene glycol) dodecyl ether, CL, cardiolipin, DAPIT, diabetes-associated protein in insulin-sensitive tissue, DDM, *n*-dodecyl-β-D-maltoside, DMNG, *n*-decyl-β-maltose neopentyl glycol, IF_1_, ATPase inhibitory factor 1, POPC, 1-palmitoyl-2-oleoyl-*sn*-glycero-3-phosphocholine, POPE, 1-palmitoyl-2-oleoyl-*sn*-glycero-3-phosphoethanolamine, POPG, 1-palmitoyl-2-oleoyl-*sn*-glycero-3-[phospho-rac-(1-glycerol)]

## Abstract

The ATP synthases have been isolated by affinity chromatography from the mitochondria of the fungal species *Yarrowia lipolytica*, *Pichia pastoris*, *Pichia angusta* and *Saccharomyces cerevisiae*. The subunit compositions of the purified enzyme complexes depended on the detergent used to solubilize and purify the complex, and the presence or absence of exogenous phospholipids. All four enzymes purified in the presence of *n*-dodecyl-β-D-maltoside had a complete complement of core subunits involved directly in the synthesis of ATP, but they were deficient to different extents in their supernumerary membrane subunits. In contrast, the enzymes from *P. angusta* and *S. cerevisiae* purified in the presence of *n*-decyl-β-maltose neopentyl glycol and the phospholipids 1-palmitoyl-2-oleoyl-*sn*-glycero-3-phosphocholine, 1-palmitoyl-2-oleoyl-*sn*-glycero-3-phosphoethanolamine, cardiolipin (diphosphatidylglycerol) and 1-palmitoyl-2-oleoyl-*sn*-glycero-3-[phospho-rac-(1-glycerol)] had a complete complement of core subunits and also contained all of the known supernumerary membrane subunits, e, f, g, j, k and ATP8 (or Aap1), plus an additional new membrane component named subunit l, related in sequence to subunit k. The catalytic domain of the enzyme from *P. angusta* was more resistant to thermal denaturation than the enzyme from *S. cerevisiae*, but less stable than the catalytic domain of the bovine enzyme, but the stator and the integrity of the transmembrane proton pathway were most stable in the enzyme from *P. angusta*. The *P. angusta* enzyme provides a suitable source of enzyme for studying the structure of the membrane domain and properties associated with that sector of the enzyme complex.

## Abstract 2

ATP, the fuel of life, is produced in mitochondria of living cells by a molecular machine, the ATP synthase. We have isolated the machines from four fungal species, compared their stabilities and identified the proteins from which they are constructed.

## INTRODUCTION

The high-resolution structural analysis of the F-ATP synthase from mitochondria has been conducted largely by the study of its constituent domains by X-ray crystallography [1–7]. The structure of the F_1_ catalytic domain [3,5,8] provides a detailed model of most of the membrane extrinsic sector of the enzyme, and a series of structures of the bovine and yeast F_1_ domains has described in detail how ATP is hydrolysed by the enzyme [5,9–14]. Other structures demonstrated that the bovine peripheral stalk, an essential component of the stator, provides a largely α-helical link between the external surface of the bovine F_1_ domain and the membrane sector of the stator, and defined its mode of attachment of the peripheral stalk to the F_1_ domain [4,6,15–17]. Structures of the F_1_–c-ring complexes from bovine [7] and yeast [2] mitochondria extended the resolved region into the membrane domain of the enzyme, and provided a description of the enzyme's rotor. It consists of the central stalk in the F_1_ domain and the tightly associated c-ring in the membrane domain. These bovine F_1_, F_1_–peripheral stalk and F_1_–c-ring substructures have been assembled into an overall mosaic structure built within the constraints of a relatively low-resolution structure of the entire bovine enzyme determined at 18 Å (1 Å=0.1 nm) resolution by cryoelectron microscopy of single particle images [18], but, as yet, there is no high-resolution structural information describing the membrane domain of the enzyme's stator. This missing region contains subunit a (or ATPase-6), the N-terminal region of subunit b and six small supernumerary subunits, e, f, g, 6.8 kDa proteolipid (known as 6.8PL) and ATP8 (or A6L) [18,19]. Subunit a is in contact with the c-ring, and together they provide the pathway for protons to traverse the membrane and generate rotation of the rotor. Because of the lack of detailed description of this interface, there remains uncertainty about how the protonmotive force is coupled to ATP synthesis. The generation of rotation also depends on an intact stator, and here subunit b has a crucial role. Its unresolved N-terminal region probably is folded into two transmembrane α-helices that interact with one or more of the five to seven predicted α-helical spans of subunit a, providing the stator with the required structural integrity. Hence a complete understanding of the mechanism of the enzyme also depends on completing the structure of subunit b.

The supernumerary subunits in the membrane domains of mitochondrial F-ATPases are so named because of both their absence from the simpler ‘core’ F-ATPases found in bacteria, and their apparent lack of direct involvement in the synthesis of ATP. Membrane subunits e, f, g and ATP8, but not subunits DAPIT (diabetes-associated protein in insulin-sensitive tissue) and 6.8PL, are conserved in *Saccharomyces cerevisiae* [20,21]. The membrane domain of the yeast enzyme also contains supernumerary subunits j and k [21], and, as described below, the new subunit l, but none of these subunits is found in the bovine enzyme. All of the yeast and bovine supernumerary subunits contain one predicted transmembrane α-helix. The bovine and yeast ATP8 subunits are oriented with their C-terminal regions in the mitochondrial matrix and their N-terminal regions in the intermembrane space [22,23]. The yeast e, f and g subunits are oriented in the opposite sense to ATP8 with their N-terminal regions in the mitochondrial matrix and their C-terminal regions in the intermembrane space of the organelle [24,25]. The N-terminal regions of bovine subunits e, f, g, DAPIT and 6.8PL are on the same side of the membrane, and their C-terminal regions are on the opposite side [26,27], and, although their absolute orientations have not been determined experimentally, it is likely that they are oriented like their yeast counterparts. The stoichiometries of supernumerary subunits in each F-ATPase complex is not known with certainty, although it is usually assumed that there is one copy of each subunit per complex.

Thus, in order to study the roles of the supernumerary subunits in the F-ATPase complex by biophysical and biochemical experimentation, there is a need to develop methods for purifying the F-ATPases with complete complements of all of their subunits. This has been achieved with the bovine enzyme by affinity chromatography using the highly specific inhibitor protein IF_1_ (ATPase inhibitory factor 1) [28]. As described in the present paper, we have sought to identify mitochondrial F-ATPases from non-mammalian sources that may be more stable, and hence more suitable for biophysical studies including structural analysis, than mammalian enzymes. We describe the isolation and the properties of the F-ATPases from the mitochondria of four fungal species, *Yarrowia lipolytica*, *Pichia pastoris*, *Pichia angusta* (also known as *Ogatea angusta* and *Hansenula polymorpha*) and *S. cerevisiae*. Each enzyme has been isolated by a rapid-affinity chromatography method on a scale that is compatible with the demands of biochemistry and biophysics.

## EXPERIMENTAL

### Analytical methods

Protein concentrations were measured using the BCA method (Pierce). The subunit compositions of purified F-ATPase complexes were analysed by SDS/PAGE in 12–22% polyacrylamide gradient gels. Proteins were stained with 0.2% Coomassie Blue dye. The ATP hydrolase activities of F-ATPases were measured by coupling the activity to the oxidation of NADH monitored at 340 nm [29]. The inhibitory effect of oligomycin on this activity was determined by addition of the inhibitor (0.1 mg/ml, w/v) in ethanolic solution.

### Fungal strains and growth

Two cultures (each 1 litre) of *P. pastoris* (strain X33) were grown for 18 h at 30°C in medium containing 1% yeast extract, 2% peptone and 2% glycerol, initially at pH 5.0. They were inoculated into medium (55 litres) of the same composition at 30°C in a 70 litre Applikon ADI 1075 fermenter, stirred at 500 rev./min with a dissolved oxygen content of 80%. The culture was grown to early stationary phase. *Y. lipolytica* strain E150 was cultivated at 27°C in a similar way in medium consisting of 2% yeast extract, 4% peptone and 4% dextrose. *P. angusta* strain A16 (NCYC 2310), a haploid leucine auxotroph of strain CBS4732, was cultivated similarly for 16 h at 40°C in 30 litres of medium containing 1% yeast extract, 2% peptone and 4% glucose, initially at pH 5.0. Cells were harvested by continuous centrifugation. The yields of wet cells for *P. pastoris*, *Y. lipolytica* and *P. angusta* were 3.6, 2.1 and 3.6 kg respectively. Cultures of *S. cerevisiae* were grown as described previously [14].

### Preparation of mitochondria

Fungal cells were suspended at a concentration of 0.5 kg/l in buffer consisting of 100 mM Tris/HCl (pH 8.0), 650 mM sorbitol, 5 mM EDTA, 5 mM aminohexanoic acid, 5 mM benzamidine and 0.2% BSA, and disrupted on ice in a Dyno-mill (Glen-Mills). During this process, the pH was maintained above 7.0 by the addition of 2 M Tris. These initial steps were performed at 18°C, and the subsequent procedures at 4°C. Cell debris was removed by centrifugation at 4000 ***g*** for 20 min, and the mitochondria were recovered from the supernatant by centrifugation at 100000 ***g*** for 30 min. They were resuspended in buffer (500 ml) containing 100 mM Tris/HCl (pH 7.5), 650 mM sorbitol, 5 mM aminohexanoic acid and 5 mM benzamidine, and centrifuged again. The pellet was resuspended in buffer (200 ml) containing 100 mM Tris/HCl (pH 8.0) and 10% glycerol, and the suspension was stored at −20°C.

### Production of inhibitor proteins

The coding sequence for residues 1–60 of the bovine inhibitor protein followed by a C-terminal GST tag and a hexahistidine tag, and, in a second construct, followed by a C-terminal decahistidine tag, were cloned into the expression vector pRUN. These inhibitors are known as bovI-(1–60)–GST–His_6_ and bovI-(1–60)–His_10_ respectively. They were overexpressed in *Escherichia coli* (DE3) strain C41 and purified as described previously for other inhibitor proteins [28].

### Purification of F-ATPases

Mitochondria (1 g) were solubilized in buffer (100 ml) consisting of 20 mM Tris/HCl (pH 8.0), 10% (w/v) glycerol and 1% DDM (*n*-dodecyl-β-D-maltoside), and containing two EDTA-free Complete™ protease inhibitor tablets (Roche Diagnostics). The F-ATPases from *Y. lipolytica* and *P. pastoris* were inhibited at 22°C with a 3-fold molar excess of bovI-(1–60)–GST–His_6_, and the enzymes from *P. angusta* and *S. cerevisiae* were inhibited at 22°C with the same molar excess of bovI-(1–60)–His_10_. The following steps were performed at 4°C. The samples of crude enzymes were centrifuged at 220000 ***g*** for 30 min and 0.15 M sodium chloride and 0.15 mM DTT (final concentrations) were added to the enzymes from *Y. lipolytica* and *P. pastoris*, and 0.15 M sodium chloride (final concentration) was added to the enzyme from *P. angusta*. The former supernatants were applied at a flow rate of 0.5 ml/min to a GST-affinity column (5 ml; GE Healthcare) and the *P. angusta* supernatant was applied at a flow rate of 0.5 ml/min to a Hi-Trap nickel–Sepharose column (5 ml; GE Healthcare). Both columns were equilibrated in column buffer containing 20 mM Tris/HCl (pH 8.0), 10% (w/v) glycerol, 0.05% DDM and 0.15 M sodium chloride and, in the cases of the enzymes from *Y. lipolytica* and *P. pastoris* only, 5 mM TCEP [tris(2-carboxyethyl)phosphine]. The *Y. lipolytica* and *P. pastoris* enzymes were released from the GST-columns by filling them with buffer (5 ml) consisting of 20 mM Tris/HCl (pH 8.0), 10% (w/v) glycerol, 0.15 M sodium chloride and 0.05% DDM plus 25 mM EDTA and 25 mM EGTA, and the columns were closed for 15 h. Then the active F-ATPases were recovered in buffer consisting of 30 mM Tris/HCl (pH 8.0), 10% (w/v) glycerol, 40 mM magnesium sulfate and 2 mM ATP. The *P. angusta* and *S. cerevisiae* F-ATPase–inhibitor complexes were released from the Hi-Trap nickel–Sepharose column by applying a gradient of imidazole from 25 to 500 mM. The inhibited complexes were released at ~150 mM imidazole. The active enzymes were recovered by addition to the inhibited complexes of 25 mM EDTA and 25 mM EGTA. After 3 h, the eluates were dialysed for 18 h against the column buffer, and the free inhibitor was removed on a nickel–Sepharose column. In some experiments, CL [cardiolipin (diphosphatidylglycerol)] (15 μg/ml) and the synthetic phospholipids POPC (1-palmitoyl-2-oleoyl-*sn*-glycero-3-phosphocholine) (50 μg/ml), POPE (1-palmitoyl-2-oleoyl-*sn*-glycero-3-phosphoethanolamine) (25 μg/ml) and POPG {1-palmitoyl-2-oleoyl-*sn*-glycero-3-[phospho-rac-(1-glycerol)]} (10 μg/ml) were added to the purification buffers. Later, the protocol for purifying the F-ATPase from *P. angusta* was modified by replacing DDM with DMNG (*n*-decyl-β-maltose neopentyl glycol). The concentration of DMNG in the buffer used for extraction of the membranes was 1% (w/v) and in the buffer used for purification was 0.05%. The enzyme from *S. cerevisiae* was purified in a similar way in the presence of the same concentrations of phospholipids and DMNG as employed for the purification of *P. angusta*.

### Characterization of the subunit compositions of fungal F-ATPases

Stained protein bands from SDS/PAGE gels were analysed by mass fingerprinting and tandem MS analysis of tryptic peptides in a 4800+ MALDI–TOF–TOF Mass Spectrometer (Applied Biosystems) and a Thermo Orbitrap XL ETD (Thermo Scientific). The masses of peptides and their partial sequences were compared using Mascot (Matrix Sciences) with a protein sequence database of the NCBI (National Center for Biotechnology Information) and against a local protein sequence database [30]. The subunits of F-ATPases were fractionated by reverse-phase chromatography as described previously [31], and the eluate was introduced ‘online’ via an electrospray interface into either a Quattro Ultima triple quadrupole instrument (Waters-Micromass) or a Q-Trap 4000 mass spectrometer (ABSciex). Both instruments were operated in MS mode, and were calibrated with a mixture of myoglobin and trypsinogen [31]. Molecular masses were calculated using MassLynx (Waters) and Bioanalyst (ABSciex). The intact purified enzymes were digested at 37°C for 16 h with trypsin (F-ATPase/protease, 50:1, w/w). The total digests were fractionated by reverse-phase chromatography and the effluent was introduced directly into an OrbiTrap mass spectrometer. The masses of selected peptides were measured. Selected ions were fragmented by collision-induced dissociation with air and a collision energy of 1 kV, and their sequences were determined by analysis of the fragments by tandem MS. The subunits of the fungal F-ATPases were transferred onto PVDF membranes by electrophoresis. Selected stained protein bands were excised and the N-terminal sequences of the mature subunits were determined by Edman degradation (Protein and Nucleic Acid Chemistry Facility, Department of Biochemistry, Cambridge University, Cambridge, U.K.).

The sequence of the ε subunit from *Y. lipolytica* was not recorded in the NCBI database. Therefore the sequences of peptides from the *Y. lipolytica* ε subunit were determined by MS, and compared using tBLASTn with protein sequences encoded in a six-phase translation of the *Y. lipolytica* genome. The genes for the δ and ε subunits of the F-ATPase from *Y. lipolytica*, and the a, δ and d subunits of the F-ATPase from *P. angusta* were resequenced. Total fungal DNA was prepared from cells of *Y. lipolytica* and *P. angusta* [32], and the genes were amplified by PCR in presence of appropriate synthetic oligonucleotide primers. The products were purified by gel purification (Qiagen), and the sequences of the amplified DNA fragments were determined by Source Bioscience, Nottingham, U.K.

Subunit k in the F-ATPase from *P. angusta* was not annotated in the genome database of the Joint Genome Institute (Department of Energy, U.S.A.). Comparison of the sequences of tryptic peptides determined by MS (see Supplementary Figure S7) with the database via Mascot detected the peptide sequences in the hypothetical protein e_gw1.3.1421.1. By comparison of its sequence using tBLASTn with protein sequences encoded in fungal genomes, subunit k was identified in *S. cerevisiae*, together with the orthologues YALI0B11913p in *Y. lipolytica* and PAS_chr3_0161 in *P. pastoris*. The sequence of a new subunit named l was identified in the preparations of *P. angusta* and *S. cerevisiae* enzymes. It was referred to previously as estExt_Genemark1.C_40616 in *P. angusta* and as YOR020W-A in *S. cerevisiae*. The sequence of the *P. angusta* l subunit was compared using tBLASTn with protein sequences encoded in the genomes of *Y. lipolytica* and *P. pastoris*

### Measurement of proton pumping by the F-ATPase from *P. angusta*

The F-ATPase from *P. angusta* was dissolved in 0.5 ml of reconstitution buffer (1 mg/ml) containing 20 mM MOPS (pH 8.0) and synthetic phospholipids (10 mg/ml; Avanti Polar Lipids). The detergent C_12_E_8_ [octa(ethylene glycol) dodecyl ether] was added to a final concentration of 0.2%, and the mixture was kept in suspension at room temperature for 10 min. Bio-beads (50 mg; Bio-Rad Laboratories) were added at hourly intervals up to 4 h. The proteoliposomes were recovered by centrifugation at 255000 ***g*** for 30 min and resuspended in the same buffer (300 μl). In the absence and presence of oligomycin (0.01%), the proteoliposomes (50 μl) were diluted with assay buffer (2.5 ml) containing 20 mM MES (pH 8.0) and valinomycin and the pH-sensitive dye ACMA (amino-6-chloro-2-methoxyacridine) were added to final concentrations of 0.4 and 0.6 μM respectively. Changes in the fluorescence of ACMA were monitored at 475 nm with an excitation wavelength of 410 nm. Proton pumping was initiated by the addition of 200 mM ATP containing 200 mM magnesium sulfate (2 μl).

### Measurement of ATP synthesis by the F-ATPase from *P. angusta*

The F-ATPase from *P. angusta* was dissolved in 0.5 ml of reconstitution buffer (0.08 mg/ml) containing 35 mM MES (pH 6.0) and a mixture of the synthetic phospholipids POPC/POPE/CL/POPG (10:5:3:2, by wt; 15 mg/ml; Avanti Polar Lipids). The inhibitor bovI-(1–60)–His_10_ (0.4 mg) was added to prevent the hydrolysis of ATP by any traces of uncoupled enzyme [29], together with the detergent C_12_E_8_ to a final concentration of 0.2%. The suspension was kept at room temperature for 10 min. Then Bio-beads (50 mg) were added at hourly intervals up to 4 h. The reconstituted proteoliposomes were recovered by centrifugation at 255000 ***g*** for 30 min, and resuspended in the same buffer (300 μl). The suspension of proteoliposomes (15 μl) was mixed with an equal volume of reconstitution buffer containing valinomycin (0.2 μM final concentration). After 5 min, it was diluted with assay buffer (270 μl) containing 300 mM HEPES (pH 8.0), 240 mM potassium hydroxide, 10 mM sodium dihydrogen phosphate, 2.5 mM magnesium chloride and 2 mM ADP. Samples (25 μl) were removed at intervals and mixed with an equal volume of trichloroacetic acid (40 g/l). They were neutralized with 2 M Tris and the ATP content was estimated using a bioluminescence assay with luciferase (Roche Biochemicals).

### Thermal inactivation of F-ATPases

Samples of purified fungal F-ATPase (2 μg) were incubated at temperatures ranging from 22 to 90°C. After 10 min, they were cooled on ice, and the residual ATP hydrolytic activities and the sensitivities of those activities to inhibition by oligomycin were measured.

## RESULTS

### Purification of F-ATPases from fungal mitochondria

**Figure 1:**
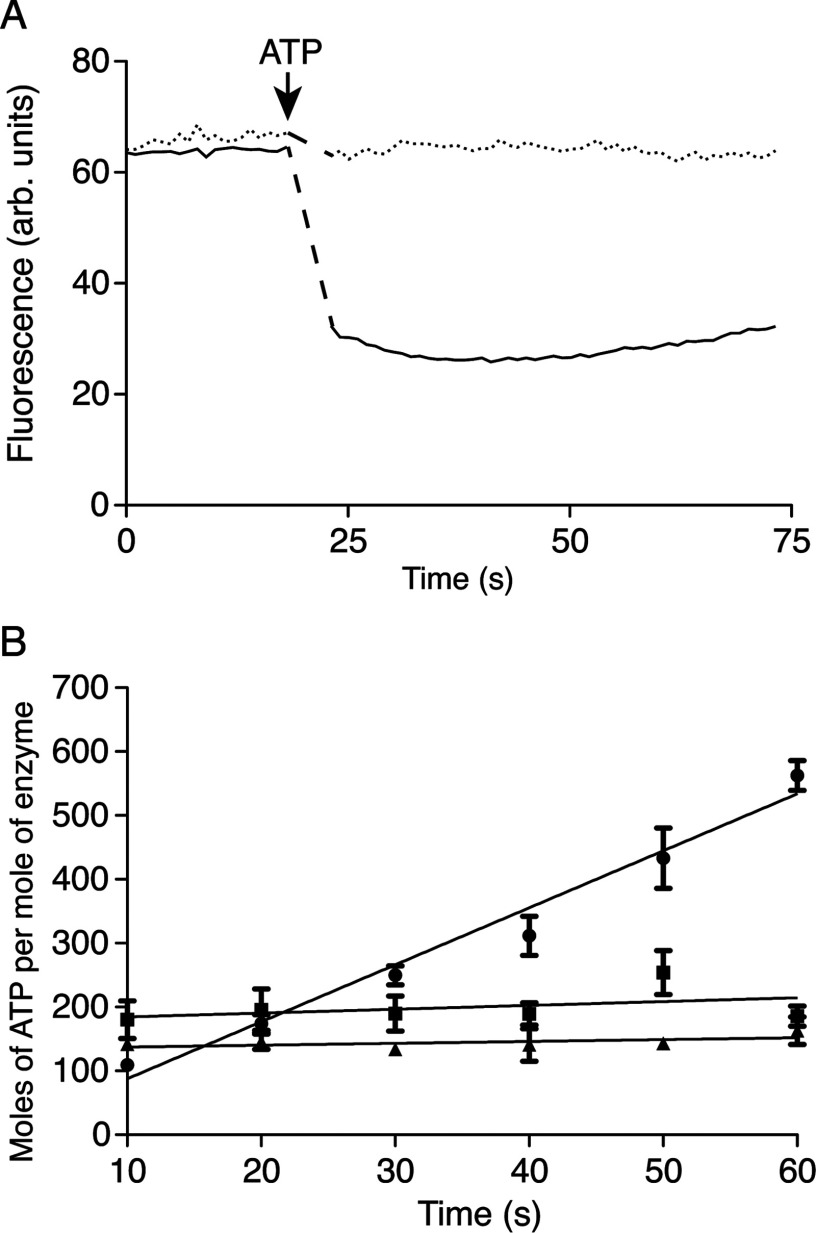
Proton-pumping activity of the F-ATPase from *P. angusta* The purified enzyme was reconstituted into liposomes. (**A**) Quenching of ACMA. The upper and lower lines correspond to the reconstituted F-ATPase from *P. angusta* in the presence and absence of oligomycin respectively. (**B**) ATP synthesis by the F-ATPase from *P. angusta* reconstituted into liposomes. Filled circles, F-ATPase only; filled squares, F-ATPase in the presence of oligomycin; filled triangles, F-ATPase in the presence of gramicidin. The rate of ATP synthesis was 8.9 s^−1^.

Initially, the fungal F-ATPases were purified by affinity chromatography with a modified version of the endogenous inhibitor protein from *S. cerevisiae*. However, bovI-(1–60) is a more potent inhibitor of the F-ATPase from *S. cerevisiae* [28], and superior yields in the range 4–8 mg of F-ATPase per g of mitochondria were obtained by replacing the yeast inhibitor with bovI-(1–60)–GFP–His_6_ for the enzymes from *Y. lipolytica* and *P. pastoris*, and by bovI-(1–60)–His_10_ for the enzymes from *P. angusta* and *S. cerevisiae*. The addition of phospholipids to the buffers employed in the purification process had no influence on the subunit compositions of the complexes, but it enhanced their ATP-hydrolytic activities and their sensitivities to oligomycin (Table 1). The presence of phospholipids in the buffers was an absolute requirement for obtaining preparations of enzyme that were capable of ATP synthesis after reconstitution into phospholipid vesicles. The most active and most highly coupled preparations of the F-ATPase were obtained from *P. angusta* and *S. cerevisiae* when the enzymes were purified in the presence of DMNG instead of DDM (Table 1). The *P. angusta* enzyme was also capable of synthesizing ATP in the presence of a protonmotive force in a reconstituted system (Figure 1). Therefore the current standard protocol for purifying the fungal enzymes is to use bovI-(1–60)–His_10_, with phospholipids and DMNG in the buffers.

**Table 1:** The ATP hydrolase activities of purified fungal F-ATPases The enzymes were purified in the absence and in the presence of synthetic phospholipids POPC/POPE/CL/POPG (10:5:3:2, by wt).

Species	Specific activity (units/mg)		Oligomycin sensitivity (%)	
Phospholipids…	−	+	−	+
Y. lipolytica*	0.2	8.4	–	92.1
P. pastoris*	2.6	9.3	37.4	95.3
*P. angusta*†	13.7	21.8	25.0	96.0
*S. cerevisiae*†	–	35.8	–	99.0

*The enzymes were purified in the presence of DDM.

†The enzymes were purified in the presence of DMNG.

### Characterization of subunits of fungal F-ATPases

**Figure 2:**
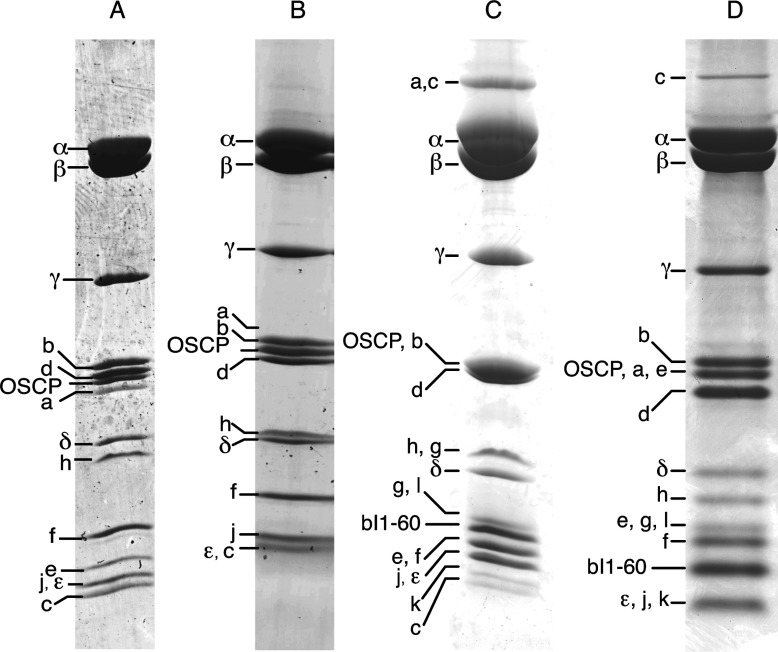
Subunit compositions of fungal F-ATPases The subunits of the F-ATPases purified from (**A**) *Y. lipolytica*, (**B**) *P. pastoris*, (**C**) *P. angusta* and (**D**) *S. cerevisiae* were separated by SDS/PAGE. They were identified by MS analysis of tryptic peptides derived from the gel bands. (**A**) and (**B**) show active enzymes; (**C**) and (**D**) show the complex of the enzyme with the inhibitor protein bovI-(1–60)–His_10_. OSCP, oligomycin-sensitivity-conferring protein.

The subunit compositions of the purified enzymes were examined by separation of the subunits by SDS/PAGE and MS analysis of tryptic digests of the stained bands (Figure 2 and Supplementary Tables S1–S3). These analyses revealed the expected complement of subunits common to mitochondrial F-ATPases from other species including *S. cerevisiae*, except for the small hydrophobic protein ATP8, which has few tryptic cleavage sites. The measurements of the masses of the intact subunits (Tables 2–4) confirmed the subunit compositions of the preparations of the three complexes and demonstrated the presence of subunit ATP8 in all three enzymes.

**Table 2:** Molecular masses of intact subunits of the *Y. lipolytica* F-ATPase OSCP, oligomycin-sensitivity-conferring protein.

Subunit*	Observed (Da)	Calculated (Da)	Δ (Da)	Modification
α	55414.9	55407.5	+7	None
β	50901.3	50894.8	+7	None
γ	30077.2	30074.2	+3	None
δ	14720.9	14719.4	+1	None
ε	6810.0	6810.8	–	None
OSCP	20519.9	20518.5	+1	None
a	27074.0	27075.3	+1	None
b	22444.8	22444.7	–	None
c	7744.6	7716.3	+28	+*N*α-formyl
d	19690.2	19820.4	−131	−Met^1^
e	9900.5	9989.2	−89	−Met^1^+*N*α-acetyl
f	10623.2	10622.5	–	None
h	9457.8	9456.0	–	None
j	6810.1	6941.0	−131	−Met^1^
ATP8	5799.9	5771.0	+29	+*N*α-formyl

*Subunits g, k and l were not found

**Table 3:** Molecular masses of intact subunits of the *P. pastoris* F-ATPase OSCP, oligomycin-sensitivity-conferring protein.

Subunit*	Observed (Da)	Calculated (Da)	Δ (Da)	Modification
α	54947.5	54839.8	+8	None
β	50883.2	50879.8	+3	None
γ	29482.4	29473.5	+9	None
δ	14508.4	14508.3	–	None
ε	6626.1	6626.6	–	None
OSCP	20497.1	20496.4	–	None
a	27609.4	27606.6	–	None
b	22271.2	22270.4	–	None
c	7832.4	7804.4	+28	+ *N*α-formyl
d	19600.7	19689.5	−89	−Met^1^+*N*α-acetyl
f	10291.6	10291.9	–	None
h	9421.9	9421.3	–	None
j	7128.9	7129.2	–	None
ATP8	5716.8	5689.1	+28	+*N*α-formyl

*Subunits e, g, k and l were not found

**Table 4:** Masses of intact subunits of the *P. angusta* F-ATPase OSCP, oligomycin-sensitivity-conferring protein.

Subunit	Observed (Da)	Calculated (Da)	Δ (Da)	Modification
α	54956	54959.0	−3	None
β	50809	50802.8	+6	None
γ	29385	29385.3	–	None
δ	14161	14161.9	–	None
ε	6899	7030.1	−131	−Met^1^
OSCP	20654	20654.9	–	None
a	27570	27550.0	+20	None
b	22267	22266.4	–	None
c	7862	7834.4	+28	+*N*α-formyl
d	19432	19522.3	−89	−Met^1^+*N*α-acetyl
e	9987	10076.5	−89	−Met^1^+*N*α-acetyl
f	9962	9962.5	–	None
g	–	15638.1		–
g	13324	13325.4		−Residues 1–21
h	9647	9647.5	–	None
j	7282	7282.7	–	None
k	6776	6776.3	–	−Met^1^
ATP8	5725	5697.1	+28	+*N*α-formyl
l	7275	7275.3	−	None

The F-ATPase from *S. cerevisiae*, purified previously by affinity chromatography in the presence of DDM [28], lacked supernumerary subunits e, g, j and k, whereas a preparation of the same enzyme purified in the presence of Triton X-100 by ion-exchange chromatography contained subunits e, g and k [24]. Similarly, the enzymes from *Y. lipolytica*, *P. pastoris* (Figures 2A and 2B respectively) and *P. angusta* (not shown), purified in the presence of DDM, lacked subunits g and k, and the enzymes from *Y. lipolytica* and *P. pastoris* additionally lacked subunit e (Figures 2A and 2B respectively). Therefore conditions for retention of these subunits were sought with the enzyme from *P. angusta*. It was found that replacing the detergent DDM with DMNG produced an F-ATPase complex that contained not only the full complement of subunits including e, g and k, but also a new supernumerary subunit, which has been named subunit l (Figure 2C).

The interpretation of the masses of the intact subunits was complicated by several of the deposited sequences being the precursors of the mature subunits in the assembled enzymes, and so they include N-terminal mitochondrial import sequences not found in the mature assembled subunits. Moreover, most of the cleavage sites for removal of the import sequences had not been determined experimentally. Although these cleavage sites could be inferred from the exact measured masses of the proteins, in many cases they were identified directly by determination of the N-terminal sequences of the mature proteins by either Edman degradation or MS sequencing of tryptic peptides (Supplementary Table S4). The N-terminal sequences of the mature subunits of fungal F-ATPases are summarized in Supplementary Figures S1–S4 and Supplementary Table S4.

An additional complication was that the masses of the δ subunit of the F-ATPase from *Y. lipolytica* and of the δ, a and d subunits from *P. angusta* measured by MS did not agree with the masses calculated from the sequence, even after taking into consideration the removal of mitochondrial import sequences, and other post-translational modifications at the N-termini of some subunits. Therefore the coding regions for these subunits were resequenced, and the deposited sequences were found to contain errors (Supplementary Figures S5 and S6). When these various corrections were taken into consideration, there was excellent agreement between calculated and measured masses of the subunits of the three F-ATPases (Tables 2–4).

Another anomaly was that the ε subunit from the F-ATPase from *Y. lipolytica* was not present in the NCBI database, although the protein was clearly a constituent of the enzyme complex. A region of homology with the gene for the ε subunit from *P. angusta* was identified in chromosome E of *Y. lipolytica*. This region was resequenced, revealing that the gene for the ε subunit is split into two exons (Supplementary Figure S5B). The characteristics of the encoded protein correspond with the MS data, which showed that the initiator methionine had been removed post-translationally.

### Recharacterization of the F-ATPase from *S. cerevisiae*

In view of enhancement of the complement of supernumerary subunits in the F-ATPase from *P. angusta* brought about by changing the detergent in buffers from DDM to DMNG, the *S. cerevisiae* enzyme was also isolated in the presence of DMNG, and its subunit composition was recharacterized by MS. The preparation of the enzyme complex now contained the same complement of supernumerary subunits as the enzyme from *P. angusta*, including the newly discovered subunit l (Figure 2D). The intact molecular masses of the subunits agreed with the calculated values (Table 5). Subunits k and l are similar in sequence, and, in order to confirm that they were indeed two distinct proteins, their individual sequences in both *P. angusta* and *S. cerevisiae* were characterized extensively (Supplementary Figure S7). These analyses confirmed that subunits k and l are orthologues, and individual genes for both subunits were identified in the genomes of *P. angusta* and *S. cerevisiae.*

Among the four fungal enzymes that were characterized, the enzyme from *S. cerevisiae* had the highest ATP hydrolytic activity, and that activity was inhibited by 99% in the presence of oligomycin (Table 1). Therefore, in this enzyme complex, ATP hydrolysis is essentially fully coupled to the generation of protonmotive force. The *P. angusta* and *P. pastoris* enzymes, when purified in the presence of phospholipids, are almost as equally well coupled as the *S. cerevisiae* F-ATPase.

### Thermal stabilities of F-ATPases

**Figure 3:**
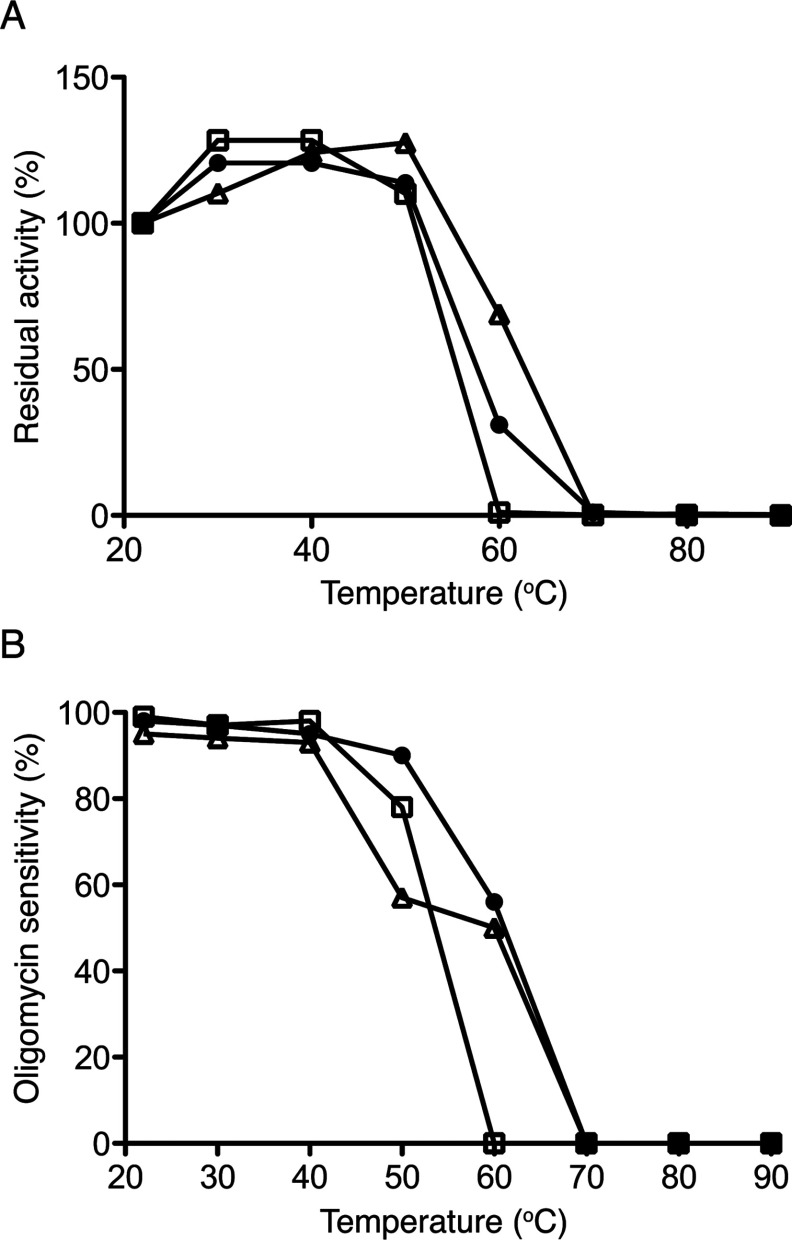
The thermal stability of purified fungal F-ATPases The enzymes were heated at a range of temperatures for 10 min. (**A**) ATP hydrolase activities of the enzymes. (**B**) Sensitivity of those activities to inhibition by oligomycin. The triangles, squares and filled circles correspond to the bovine enzyme and the enzymes from *S. cerevisiae* and *P. angusta* respectively.

The impact of heating on the activities of the F-ATPases purified from bovine mitochondria and from the mitochondria from *S. cerevisiae* and *P. angusta* is shown in Figure 3. The measurements of the hydrolysis of ATP (Figure 3A) demonstrate the effect of heating on the enzymic activity of the F_1_ catalytic domain. The measurement of the sensitivity of the hydrolytic activity to inhibition by oligomycin (Figure 3B) provides a measure of the effect of heating on both the catalytic activity of membrane extrinsic F_1_ domain and on the coupling of ATP hydrolysis to the proton-pumping activity of the enzyme. The coupling of the enzyme depends on the integrity of the stator, including the catalytic sites and the peripheral stalk, and the maintenance of the transmembrane proton pathway, which itself requires the maintenance of contact between the a subunit in the stator and the c-ring in the rotor. As shown in Figure 3(A), all three enzymes remained active up to 50°C and their ATPase activities were stimulated by increased temperature. At higher temperatures, ATP hydrolysis activity was progressively lost from all three enzymes. The yeast enzyme was the least resistant, and the bovine F-ATPase was marginally more stable than the enzyme from *P. angusta*. Similar trends are evident in Figure 3(B), although oligomycin sensitivity was not stimulated by increased temperature, but full sensitivity to the inhibitor was maintained up to 50°C. At higher temperatures, the sensitivity to oligomycin survived best with increasing temperature in the enzyme from *P. angusta*, indicating that its stator, including the a subunit plus the contact of the a subunit with the c-ring was the most stable. In this respect, it is significant that the enhanced stability of the a–c oligomer from *P. angusta* is manifest from its persistence under the conditions of denaturing gel electrophoresis (Figure 2C).

**Table 5:** Masses of intact subunits of the *S. cerevisiae* F-ATPase OSCP, oligomycin-sensitivity-conferring protein.

Subunit	Observed (Da)	Calculated (Da)	Δ (Da)	Modification
α	54951	54944.7	+6	∆ import
β	51135	51126.4	+9	∆ import
γ	30619	30616.2	+3	∆ import
δ	14553	14553.5	–	∆ import
ε	6611	6611.4	–	−Met^1^
OSCP	20870	20871.2	−1	∆ import
a	27952	27939.7	+13	−residues 1–10
b	23166	23165.6*	–	∆ import
	23035		−131	∆ import; −Met^1^
c	7787	7759.4	+28	+*N*α-formyl
d	19720	19809.6	−89	−Met^1^+*N*α-acetyl
e	10786	10875.5	−89	−Met^1^+*N*α-acetyl
f	10565	10565.2	–	None
g	12921	12921.2	–	None
h	10408	10407.4	–	None
i	6687	6687.8	–	None
k	7402	7533.8	−131	−Met^1^
aap1	5850	5850.3	–	None
l	9486	9617.7	−131	−Met^1^

*R137A variant in the mature amino acid sequence [31].

## DISCUSSION

### Purification of F-ATPases by affinity chromatography

The purification and study of F-ATPases from mitochondria has been aided greatly by the development of a simple affinity chromatography method that exploits the exquisite selectivity of the natural inhibitor protein IF_1_ for mitochondrial F-ATPases [28]. However, IF_1_ does not inhibit the bacterial and chloroplast F-ATPases, and therefore the F-ATPases from these sources usually require the introduction of an appropriate covalent affinity tag into one of the subunits, where this is possible, or, alternatively, they have to be purified by classical chromatographic procedures. The affinity method for mitochondrial enzymes was applied initially to the purification of the enzyme from the mitochondria of cows, sheep, pigs and *S. cerevisiae* [28], and then it was extended to the F-ATPases from other mammals, reptiles, birds, amphibians, ray-finned fish and invertebrates [33]. In the present study, it has been demonstrated that the method can be applied with success to a range of fungal species. Somewhat surprisingly, the bovine IF_1_ has a greater affinity for the yeast F-ATPase than the yeast orthologue [28], and therefore derivatives of bovine IF_1_ are preferred for purifying the fungal enzymes. The utilities of the inhibitors bovI-(1–60)–GST–His_6_ and bovI-(1–60)–His_10_ were compared. The former was employed for purifying the *Y. lipolytica* and *P. pastoris* enzymes via the interaction of its GST domain with immobilized glutathione and the latter for purifying the *P. angusta* and *S. cerevisiae* enzymes. The former gave highly pure enzyme, but with relatively low yields. Therefore the latter procedure, which produces pure enzyme with relatively high yields is preferred for most purposes.

Initially, as described previously for the enzyme from *S. cerevisiae*, the enzymes from *Y. lipolytica* and *P. pastoris* were purified in the presence of the detergent DDM and in the absence and in the presence of phospholipids (Table 1 and Figures 2A and 2B). The experiments confirmed that the presence of phospholipids enhanced both the specific activity of the enzyme and its sensitivity to oligomycin. It also became apparent that the replacement of the detergent DDM by another detergent, DMNG, improved both the activity of the enzyme and the integrity of the proton pathway, as indicated by the improvement in sensitivity of the hydrolytic activity to oligomycin (Table 1). As described in the following section, it also had an impact on the subunit composition of the purified enzyme complex.

### The subunit compositions of fungal F-ATPases

**Figure 4:**
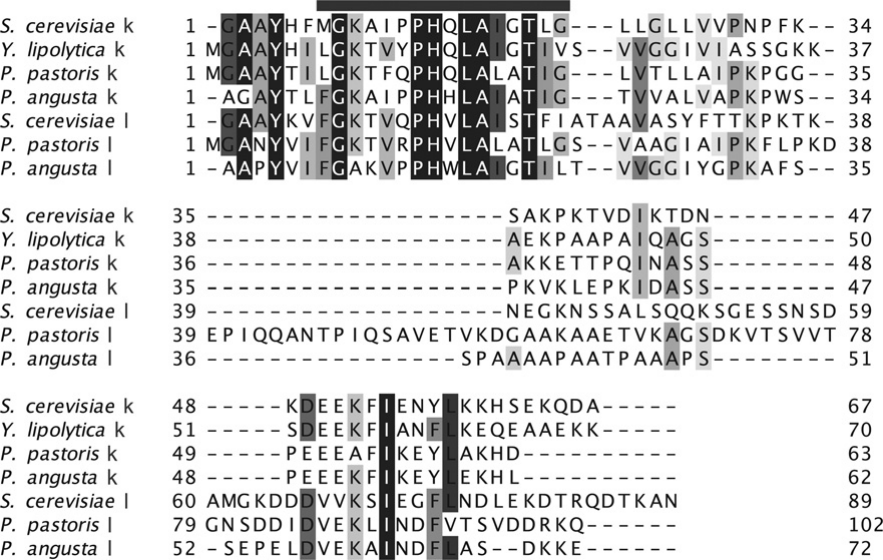
Alignment of the sequences of fungal subunits k and l The N-terminal methionine residues in the k and l subunits from *P. angusta* and *S. cerevisiae* are removed post-translationally. In *Y. lipolytica* and *P. pastoris*, the protein has not been characterized. There is no subunit l in *Y. lipolytica*. A predicted transmembrane α-helix is indicated by a black bar.

The extensive MS analyses conducted on the purified fungal F-ATPases uncovered deficiencies in the annotation of the subunits of the enzymes, and in the deposited sequences of some subunits. These deficiencies have been rectified. They also showed that when the enzymes were purified in the presence of DDM, the F-ATPases from *Y. lipolytica* and *P. pastoris* lacked supernumerary subunits g and k, and the enzyme from *P. pastoris* additionally lacked supernumerary subunit e. Similar results to those obtained with the *P. pastoris* enzyme were obtained with the enzyme from *P. angusta* (not shown). However, replacing the detergent DDM with another detergent, DMNG, in the purification of the enzyme from *P. angusta* produced a purified complex that contained not only the known supernumerary subunits ATP8, e, f, g, j and k, but also a new subunit, named l, that had not been detected previously in the enzyme from *S. cerevisiae*, or in any other fungal F-ATPase. When the enzyme from *S. cerevisiae* was re-isolated in the presence of DMNG and its subunit composition was recharacterized, it had the same complement of subunits as the enzyme from *P. angusta*, including subunit l. No attempt has been made to verify their presence experimentally in the *Y. lipolytica* and *P. pastoris* F-ATPases, but the presence of orthologues in both genomes (see Figure 4) indicates that they are probably constituent subunits of the F-ATPases in those species also. The sequences of subunits k and l are 18% identical in *S. cerevisiae*, 38% identical in *P. pastoris* and 31% identical in *P. angusta*, and are most closely related in their N-terminal regions (Figure 4).

### Roles of supernumerary subunits

Some of the supernumerary subunits of the mitochondrial F-ATPases have defined structural roles in the enzyme complex. Thus, despite extensive differences in the length and sequence of their polypeptide chains, bovine and yeast ATP8 subunits appear to be essential components of the peripheral stalk regions of the enzymes [27]. In their absence, or if the protein is truncated or mutated in its C-terminal region, the enzyme is uncoupled or it fails to assemble [34]. In the cristae in the inner membranes of mitochondria, the F-ATPase complexes are dimerized via interaction of the membrane domains of the monomers, and the dimers are arranged in rows along the edges of the cristae [35]. In yeast mitochondria, subunits e and g are involved in the formation of the dimers, and deletion of subunit e changes the structure of the inner membranes dramatically [35]. It is likely that subunits e and g have a similar role in F-ATPases in other species. Yeast subunits e and g have also been proposed to play a role in the mitochondrial permeability transition pore [36], which is involved in the process of triggering cell death by necrosis [37,38]. The roles of the remaining supernumerary subunits, g, DAPIT and 6.8PL in the bovine enzyme, and g, j, k and l in the fungal enzymes, remain to be elucidated.

### Further characterization of mitochondrial F-ATPases

The advantages of enhanced protein thermostability were recognized first in structural studies of glycolytic enzymes and aminoacyl-tRNA synthetases from the moderate thermophile *Bacillus stearothermophilus* (now *Geobacillus stearothermophilus*) [39–41], and the enhancement of thermostability by mutation has been employed more recently, for example, to facilitate structural studies of G-protein-coupled receptors [42,43]. Hence, the primary aim of the present study was to identify a eukaryotic source of an F-ATPase with enhanced stability, relative to the bovine enzyme. *P. angusta* can be grown at temperatures up to ~50°C and is classified as a moderate (or facultative) thermophile. *P. pastoris* and *Y. lipolytica* were included in the study for comparison, and *S. cerevisiae* because the enzyme has been studied extensively, and its sophisticated genetic systems offers the opportunity for a wide range of additional experiments. *Y. lipolytica*, *P. pastoris* and *S. cerevisiae* are mesophilic organisms with optimum growth temperatures in the range 25–30°C. These growth temperatures are reflected in the observed thermal inactivation of their F-ATPases. The enzyme from *P. angusta* was more thermostable than the enzymes from either *Y. lipolytica* or *P. pastoris* (results not shown). Moreover, the sensitivity of its ATP hydrolytic action was also more resistant to thermal inactivation than that of the F-ATPase from bovine or yeast mitochondria. Therefore, for biophysical studies, including structural analysis of its membrane domain, required for the structural elucidation of the transmembrane proton pathway and the mitochondrial permeability transition pore, the *P. angusta* enzyme appears to be the enzyme of choice. The determination of the stoichiometries of its supernumerary subunits and the characterization of the oligomeric state of the purified enzyme are necessary preludes to such experiments, and they are being studied currently.

## Online data

Supplementary data
