# Supplementary material for: The purification and characterization of ATP synthase complexes from the mitochondria of four fungal species
Source: Biochem J. 2015 May 5;468(Pt 1):167–75. doi: 10.1042/BJ20150197 (PMC4422255; doi:10.1042/BJ20150197)
Supplement: Supplementary data [file bj4680167ntsadd.pdf]

Supplementary information for:

The purification and characterization of F-ATPases from the mitochondria of four fungal species

Sidong LIU, Thomas J. CHARLESWORTH, John V. BASON, Martin G. MONTGOMERY, Michael E. HARBOUR, Ian M. FEARNLEY and John E. WALKER

The Medical Research Council Mitochondrial Biology Unit, Cambridge Biomedical Campus, Hills Road, Cambridge CB2 0XY, United Kingdom

[1]To whom correspondence should be sent: walker@mrc-mbu.cam.ac.uk.

Table S1 Summary of peptide mass fingerprinting and tandem MS data for the F-ATPase from *Y. lipolytic*a

	Peptide mass fingerprinting			Tandem MS	
Subunit[1]	MASCOT score	Peptides (n)	% coverage	Peptides (n)	Score[2]
α	696/82	36	53	7	560/53
β	740/82	31	65	7	498/54
γ	658/82	25	46	6	542/54
δ	-	-	-	2	81/50
ε	-	-	-	3	-[3]
OSCP	614/82	18	74	5	483/53
a	293/82	10	22	3	233/54
b	416/82	17	63	2	128/53
c	-	-	-	1	55/49
d	653/82	17	63	6	510/53
e	217/82	7	52	1	146/53
f	435/82	16	44	4	263/53
h	110/82	2	20	1	101/51
j	-	-	-	2	139/51

[1] In these experiments, no evidence was found for subunits g, k, l and ATP8; [2] the score is the sum of significant ions scores for individual peptides where each ions score is above the p=0.05 confidence limit. [3]The sequence of the ε-subunit is not recorded in NCBI database; peptides were identified manually.

Table S2 Summary of peptide mass fingerprinting and tandem MS data for the F-ATPase from *P. pastoris*

	Peptide mass fingerprinting			Tandem MS	
Subunit[1]	MASCOT score	Peptides (n)	% coverage	Peptides (n)	Score
α	623/50	32	51	7	437/23
β	602/50	29	69	6	321/23
γ	505/50	17	33	5	454/23
δ	111/22	3	32	4	321/23
ε	66/50	3	36	2	167/23
OSCP	169/50	9	59	3	150/21
a	-	-	-	1	53/23
b	406/50	14	46	8	483/23
c	-	-	-	1	- [2]
d	485/50	20	63	7	421/23
f	115/83	5	20	3	241/26 [3]
h	100/50	2	23	1	90/22
j	55/50	6	25	1	31/26

[1] In these experiments, no evidence was found for subunits e, g, k, l and ATP8; [2] as the peptide identified was the αN-formyl N-terminal tryptic peptide, there was no match to the sequence database and no MASCOT ions score; [3] data from analysis of chymotryptic peptides.

Table S3 Summary of peptide mass fingerprinting and tandem MS data for the F-ATPase from *P. angusta*

	Peptide mass fingerprinting			Tandem MS	
Subunit[1]	MASCOT score	Peptides (n)	% coverage	Peptides (n)	Score
α	505/53	12	31	2	147/57
β	634/53	18	36	4	309/57
γ	93/53	10	53	7	447/57
δ	296/85	2	42	2	343/57
ε	37/53	1	36	1	37/31
OSCP	410/85	6	42	3	380/58
a	-	-	-	2	155/30
b	374/85	6	30	4	332/58
c	-	-	-	1	107/58
d	470/85	6	40	4	386/58
e	299/88	3	53	3	348/60
f	179/85	4	65	2	128/60
g	60/53	3	34	2	101/57
h	-	-	-	2	288/29
j	50/53	1	26	1	119/25
k	155/53	2	57	1	113/26
l	-	-	-	4	180/- [2]

[1] In these experiments, no evidence was found for subunit ATP8; [2] tandem MS data for the l-subunit were compared with a custom sequence database containing too few sequences to produce a reliable p=0.05 confidence limit.

A

```
S. cerevisiae    1 ASTKAQPTEVSSILEERIKGVSDEANLNETGRVLAVGDGI 40
Y. lipolytica   25 AEAKATPTEVTSILEERIRGVSGEANLNETGRVLSVGDGI 64
P. pastoris     37 AAAKAAPTEVSSILEEKIRGVSAEADLNETGRVLSVGDGI 76
P. angusta      62 ATAKAAPTEVSSILESKIRGVSDEANLDETGRVLSVGDGI 101
```

B

```
S. cerevisiae    1 ASAAQSTPITGKVTAVIGAIVDVHFEQSELPAILNALEIK 40
Y. lipolytica   28 --ASSAGVGSGKIRTVIGAVVDVQFEQDNLPAILNALTID 65
P. pastoris     29 ---ATAAASTGKVRAVIGAVVDVQFEQGQLPDILNALELQ 65
P. angusta      28 ---ATAGPASGKIRAVIGAVVDVQFEQGELPAILNALTID 64
```

C

S. cerevisiae	1	ATLKEVEMRLKSIKNIEKITKTMKIVASTRLSKAEKAKIS	40
Y. lipolytica	22	ATLREIEMRLKSIKNIEKITNTMKIVASTKLGKAQRAMAT	61
P. pastoris	21	ATLREIEMRLKSIKNIEKITNTMKIVASTRLNKAQRAMAT	60
P. angusta	19	ATLREIETRLKSIKNIEKITNTMKVVASTRMGRAQRAMAS	58

D

S. cerevisiae	1	AEAAAASSGLKLQFALPHETLYSGSEVTQVNLPAKSGRIG	40
Y. lipolytica	25	AETAS-VDKLRLTLALPHQSIYNQKEVTQVNIPSTAGELG	63
P. pastoris	22	AEAAATSDVLKVSLALPHETIIDNKEAAQVNLPGASGELG	61
P. angusta	24	AEAVN-PDVLKVSLVAPHQAIFTNKEVSQVNLPASSGEMG	62

E

S. cerevisiae	1	-SAWRKAGISYAAYLNVAAQAIRSSLKTELQTASVLNRSQ	39
Y. lipolytica	1	MSAWKSAGFSFNKYSAIAARTVRKALTEQKR-AHVNARDL	39
P. pastoris	2	-SSWQKAGFSFNKYLSIAARTVRKSLNSDIR-VAAEKRGI	39
P. angusta	2	-SSWQKAGISFNKYLAIAARTVQRSLKNDLK-VAAEKRYI	39

Figure S1 Alignment of N-terminal sequences from subunits of the F_1-domain of F-ATPases from fungi

Parts A-E, the N-terminal sequences of the mature α, β, γ, δ subunits and ε-subunits. In this Figure and in Figures S2-S4, dark blue and light blue areas indicate identical and conservatively substituted residues, respectively. For experimental verification of N-terminal regions, see Table S4.

A

S. cerevisiae	1	ASKAA--APPPVRLFGVEGTYATALYQAAAKNSSIDAAFQ	38
Y. lipolytica	20	ATKAAAPVKVPVQLFGLDGTYATALYTAAAKESDLSKTEG	59
P. pastoris	11	ATAAKT-VKPPVQLFGLDGTYATALYTASVKVSSVDQAAG	49
P. angusta	12	ASAAP--IKPPVQLFGLDGTYATALFSASAKDSSIEKTFQ	49

B

S. cerevisiae	1	MSSTPEKQTDPKAKANSIINAIPGNNILTKTGVLGTSAAA	40
Y. lipolytica	15	-----SNQVDPKVKATSILDSIPGNNVLSKTGVLATGVLG	49
P. pastoris	29	----QSSQVEPKAKANSIIDALPGNNILSKTGIVATSVAG	64
P. angusta	1	--STP---VDPKTKANALIDSLPGNSFLSKTGILATTAAA	35

C

S. cerevisiae	1	--SLAKSAANKLDWAKVISSLRITGSTATQLSSFKKRNDE	38
Y. lipolytica	2	SVAAARSSAVKVDWGKIVSSLGLTGATVSSLQAFRKRHEE	41
P. pastoris	2	-SSVAKQAATKLDWATIISKLGLTGSTAASLTSFKKRHDE	40
P. angusta	2	-SSVAKSTANKLDWTKIVSKLGLSGQTAAALTSFKKRNDE	40

D

```
S. cerevisiae    1 NVIQDLYLRELKDTKLAPSTLQDAEGNVKPWNPPQKPNLP 40
Y. lipolytica   20 NLISEAYTREVRAFKAPKLSAKDAEGQVKPWSAPSAPKPP 59
P. pastoris     25 NLISDLYVKELKGFKPTPLSAADAEGATKPWAKPASPKVP 64
P. angusta      28 NLVTDLYVKELKAFKPTPASAADAEAATKPWKLPQAAKVP 67
```

Figure S2 Alignment of the N-terminal sequences from the mature subunits of the peripheral stalk region of the F-ATPases from fungi

Parts A-D, the N-terminal sequences of the OSCP, b. d subunits and h subunits. The N-terminal residues of d subunits are acetylated. In the h subunit from *P. pastoris*, the observed N-terminal residue was aspartic acid, not asparagine.

A

```
S. cerevisiae    1 SPLDQFEIRTLFGLQSSFIDLSCLNLTTFSLYTIIVLLVI 40
Y. lipolytica    7 SPLEQFTTRVYFGLSSGLINLDTITLTSFSIYSIAVVALI 46
P. pastoris     14 ----QFDMKLFMGFASPMDFMSNLNMTTFTMYTMLVYLVM 49
P. angusta       8 SPLDQFIINNLLEINSPFLNLSTLNFSTFSLYTLFVVLVI 47
```

B

```
S. cerevisiae    1 MQLVLAAKYIGAGISTIGLLGAGIGIAIVFAALINGVSRN 40
Y. lipolytica    1 MQLVLAGKYIGAGLASIGLVGAGIGIAIVFAALINGVSRN 40
P. pastoris      1 MQLVLAAKYIGAAIATIGLTGAGIGIAIVFAALINGTSRN 40
P. angusta       1 MQLVLAAKYIGAAIATIGLTGAGIGIAIVFAALINGTSRN 40
```

C

```
S. cerevisiae    1 MPQLVPFYFMNQLTYGFLLMITLLILFSQFFLPMILRLYV 40
Y. lipolytica    1 MPQLVPFYFTNQIFYGFASLSVIVYLFSIYILPHYLEIYV 40
P. pastoris      1 MPQLVPFYFLNQLTYGLLLIYLILVLVSTKILPYIIELYI 40
P. angusta       1 MPQLIPFFFLNQLFYGYLALFALLVLVSWVILPYLLQLQI 40

S. cerevisiae   41 SRLFISKL                                 48
Y. lipolytica   41 TRIFITKT                                 48
P. pastoris     41 IRMTISKL                                 48
P. angusta      41 VRLLITKL                                 48
```

Figure S3 Alignment of sequences from the mature mitochondrially encoded membrane subunits of the F-ATPases from fungi

Parts A and B, N-terminal sequences of the mature a and c subunits; part C, the entire ATP8 subunits. The N-terminal residues of the c and ATP8 subunits are formylated.

A

```
S. cerevisiae    1 STVNVLRYSALGLGLFFGFRNDMILKCNAKKEEQAQYEE  40
Y. lipolytica    2 STLNVLRWSALGAGVVYGFVHNRTLYSQAEKKVADAKFKK 41
P. pastoris      2 STLNVLRYSSLAAGIVYGAYHTYTLKLEGEKKQELYDYQK 41
P. angusta       2 STLNVLRYSSLAAGIVYGAYHTYTLKLEGEKKQELYDYQK 41

S. cerevisiae   41 KLKLVEEAKKEYAKLHPVVTPKDVPANASFNLEDPNIDFE 80
Y. lipolytica   42 QEKLIEQAKAEWARLHP---APVASTGVVTDISDDKFDIE 78
P. pastoris     42 KLKLVEAAKAEYRKLNP---PKQAASTEAVNFDDPEFDFG 78
P. angusta      42 KLKLVEAAKAEYRKLNP---PKQAASTEAVNFDDPEFDFG 78

S. cerevisiae   81 RVILNAVESLKEAST                          95
Y. lipolytica   79 AYLNHAFPEKA----                          89
P. pastoris     79 KFILGAVEKLG----                          89
P. angusta      79 KFILGAVEKLG----                          89
```

B

```
S. cerevisiae    1 VSTLIPPKVVSSKNIGSAPNAKRIANVVHFYKSLPQGPAP 40
Y. lipolytica    7 LSTLIPPKVASPATLHGAPNAKRMADVVSFYKALPQGAAP 46
P. pastoris      9 LSTLIPPKIASAKNLGSNPHAKKIQEVVKFYKALPQGQAS 48
P. angusta       9 LTTLIPPKVASASNLGSNPAAKRMQHIVSFYSKLPRGEAS 48

S. cerevisiae   41 -AIKANTRLARYKAKYFD--GDNASGKPLWHFALGIIAFG 77
Y. lipolytica   47 ALPKTANPFKLYYRKYFHPKSGKASGAPLLHLILGIFLFG 86
P. pastoris     49 -FPKASSPIGKYGEKYFN--DGNASAKPLLHLALGVILFG 85
P. angusta      49 -FPKAKSPLGLYREKYFD--TG--SGAPLLHASLFFLAVG 83

S. cerevisiae   78 YSMEYYFHLRHHKGAEEH                       95
Y. lipolytica   87 YISDYQFHLKHHKNG-AH                       103
P. pastoris     86 YSLEYYYHLRHHKG--EH                       101
P. angusta      84 YGLEYYFHLSHHK---EH                       98
```

C

```
S. cerevisiae    1 ------------------MLSRIQNYTSGLVSKANLLSSK 22
Y. lipolytica    1 MFRSRVSGVFQQVRFQSTAASKAASKAQGLGAKVQGITNC 40
P. pastoris      1 -MLSRSLIQSTKTATRNVRFASTNASKQSIVEKVTNLSSQ 39
P. angusta      22 -------------------------ISQYIT-KAQGFFNQ 35

S. cerevisiae   23 ALYYGKVGAEISKQIYLKEGLQPPTVAQFKSVYSNLYKQS 62
Y. lipolytica   41 AVYWAKVTGELGKQIYLKEGFAPPSLSQFQSVYQNLFNSV 80
P. pastoris     40 VVYWGKVTGEIAKQVYVKEGLSPPTTTQIQSVYQDLYKKA 79
P. angusta      36 AIYWTKVTVEVSKQIYIREGLAPPSVAEIQQVYQGLYKKA 75

S. cerevisiae   63 LNFALKPTE----VLSCLKNIQKNELLKYGAYGIQLIGFY 98
Y. lipolytica   81 KSYALKPQK----VIDCAESITKTDALRYTAYGVQILGLF 116
P. pastoris     80 LESFANPQAAFQSVKESAKNLNKDIVLKYGAYGIQLVGLF 119
P. angusta      76 LEFAAQPKTSADGLIKVAKSLSKEEYLRFGAYFIQIVGLF 115

S. cerevisiae   99 SVGEIIGRRKLVGYKHH---------               115
Y. lipolytica  117 TLGEVIGRRNVIGYKVPSADKH---                138
P. pastoris    120 SLGEIIGRRQIVGYPSFGPKAAAHH                144
P. angusta     116 SLGEIIGRRQIVGYPSFGPKEHHH-                139
```

D

```
S. cerevisiae    1 ----MLKRFPTPILKVYWPFFVAGAAVYYGMSKAADLSSN 36
Y. lipolytica    2 -AFGIRRAYPTPIVKPLWPYAVGGVITFFLFAKAANASMN 40
P. pastoris      2 ---VNVTRYAVPILKPYWTFFAGAGITYYLIGKAANASMN 38
P. angusta       1 MKFLGVKVYRFPLVKYYWPFFVGFGLTFYGVAKIQNAMMD 40

S. cerevisiae   37 TKEFINDPRNPRFAKGGKFVEVD---               59
Y. lipolytica   41 TEEFINDPRNPRFKAGG--VKEEH--               62
P. pastoris     39 SDEFINDPRHPRFNRGEKVIDIKALP               64
P. angusta      41 TAEFINDPRHPRFKKGD--LEKK---               61
```

Figure S4 Alignment of the sequences from nuclear encoded supernumerary membrane subunits of the F-ATPases from fungi

Parts A-D, the sequences of the entire mature e, f, g and j subunits, respectively. The N-terminal serine residue in e-subunits is acetylated. The alignments of the k and L subunits are presented in Figure 4.

Table S4 Amino-terminal sequences of subunits of fungal F-ATPases

Species	Subunit	Sequence
Y. lipolytica	α	AEK [1]
	β	ASSAGV [3]
	γ	ATLREI [3]
	δ	AETASVDKLR [2]
	ε	MSAWKS [3]
	OSCP	ATKAAA [3]
	a	SPLEQF [3]
	b	SNQVDP [3]
	c	Nα-formyl MQLVLAGK[2]
	d	SVAAAR [3]
	e	Nα-acetyl STLNVLR[2]
	f	LSTLIP [3]
	h	NLISEA [3]
	j	AFGIRR [3]
	ATP8	Nα-formyl MPQLVP [3]
P. pastoris	α	AAAXAAP [1]
	β	ATAAAS [1]
	γ	ATLRE [1]

	δ	AEAAATS [1]
	ε	SSWQKAGISFNKYLAIAARTVQR[2]
	OSCP	ATAAKTV [1]
	a	QFDMKL [3]
	b	XSSQVEP [1]
	d	Nα-acetyl SSVAKQ [3]
	c	Nα-formyl MQLVLAAK [2]
	f	LSTLIPP [1]
	h	DLIS [1]
	j	VNVTRY [3]
	ATP8	Nα-formyl MPQLVP
P. angusta	α	ATAKA [1]
	β	ATAG [1]
	γ	ATLREIE [1]
	δ	AEAVNDDV [1]
	ε	SSWQKAGISFNKYLAIAAR [2]
	OSCP	ASAAPIKPPVQLFGLDGTYATALFSASAK[2]
	a	SPLDQFIINNLLEINSPF [2]
	b	STPVDPK [3]
	d	Nα-acetyl SSVAKSTANK [2]
	c	Nα-formyl MQLVLAAKY [2]
	e	Nα-acetyl STLNVLR [2]
	f	LTTLIPPK [2]
	g	ISQYITK [2]
	h	NLVTDLYVKE [1]
	j	MKFLGVK [2]
	k	AGAYTLFGK [2]
	l	AAPYVIFGAK [2]
	ATP8	Nα-formyl MPQLIP [3]

Footnotes: [1] by Edman degradation; X, unidentified amino acid; [2] by tandem MS of N-terminal tryptic peptides; [3] inferred only from the measured mass of the intact protein. In each case, residues 1-6 of the mature protein are shown.

A

```
AACACGACACCTTGCACTAACAACAACACAATGTTTTCTGTTGCTCGAACCGCCATCCGG
                              M  F  S  V  A  R  T  A  I  R
GGTGCTGCCCGACCCGCCGTGCGAATTGCTCGACGAGGTTACGCCGAGACTGCTTCCGTC
G  A  A  R  P  A  V  R  I  A  R  R  G  Y  A  E  T  A  S  V
GACAAGCTTCGACTGACTCTCGCTCTTCCCCACGAGGTGAGTAATGTGGTGATGTGCTAA
D  K  L  R  L  T  L  A  L  P  H
CGACATGGAGGACGGGATGACACAGGCGACTGAGAACGTCCAGCGACACTGAGGAACCGA

GACAATTGGTAGAAAATGACGACACAATTGAGGCTGCAATGCGAATTGACTGGGACAGAG

GACATATAGTAGCGTGGTTTATTCGGTGTACCGAGTTATACAGACCGGTCAAGTCTGTGT

GATGCGGTGGTGGTGGGAGGGAAGGAACGTGACTTGGCATGGCATGGCGTGGCGTGGCGC

GTGGACTCTGCATGGTGGTGGAATTGGGTCGCAGGCAGCCGACTGGTCAATTGACCTTGT

TTGTGCTCATGATCCACGCCACTGCCGGAAGCACAAGGAAACGCGGGAGACATCACCTCA

CATCACGACACCGTACCAACCACCCAATGTACAATGAACCACTCGCGAATTGAAGCTCCT

CAAATGTTGTGATACCCAGCATTCCCTTTTGGCTCTGTTGCTGTCGACCCCAGGCGCCAG

TATGTTCCAGTCGCGATGTCCGATCACACAAGGTCACAAAACTGGACGAACCTCATACTA

ACTCAGTCCATCTACAACCAGAAGGAGGTGACCCAGGTCAACATCCCTTCCACCGCTGGT
   Q  S  I  Y  N  Q  K  E  V  T  Q  V  N  I  P  S  T  A  G
GAGCTCGGTATTCTGGCTAACCACGTTCCCACCATCCAGCAGCTCAAGCCTGGTGTTGTC
E  L  G  I  L  A  N  H  V  P  T  I  Q  Q  L  K  P  G  V  V
GAGGTCATTGAGACTAACGGTGAGACCAAGTCTTACTTCATCTCCGGTGGTTTCGCTACC
E  V  I  E  T  N  G  E  T  K  S  Y  F  I  S  G  G  F  A  T
GTTCAGCCCGACTCCGAGCTGTCCGTCAACTCCATTGAGGCTTTCCAGGCTGAGGACTTC
V  Q  P  D  S  E  L  S  V  N  S  I  E  A  F  Q  A  E  D  F
TCCCCCGAGGCCATCAAGTCTCTGACCGCCGAGGCCCAGAAGAACGCCCAGTCTGCTGAC
S  P  E  A  I  K  S  L  T  A  E  A  Q K  N  A  Q  S  A  D
GAGGCTGTTGCTGCTGAGGCCGAGATTGAGCTCGAGGTTCTTGAGGCTCTTGCCCACTTT
E  A  V  A  A  E  A  E  I  E  L  E  V  L  E  A  L  A  H  F
GCCAAATAAGTTATGACATGATGAATCTGTAGACATAAAAGCGGCAACTTCCGTAACGTG
A  K
```

B

```
TTCTCAGAAAACAACACCACACAATGTCTGCCTGGAAGTCTGCCGGTTTTTCGTGAGTACCAT
                       M  S  A  W  K  S  A  G  F  S
CGAACGGGGTGCAATGACTCGCCAATTGCGCTGATGGACTGATTGCGTTCCGAATTGCTGTTT

TTTCGGCTGTCCGACCCATGTCCGACTCATGTCCATGAACACCAGCACCCACGACACAGCACA

CATTAGCGATTCAAAGACACATACTAACCCAGCTTCAACAAGTACTCTGCCATTGCTGCTCGA
                                       P  S  F  N  K  Y  S  A
ACCGTCCGAAAGGCCCTTACCGAGCAGAAGCGAGCCCATGTCAACGCCCGAGATCTCTCTGAG

ATTAAGGTCGCCAAGTGGGAGTCTGGAAAGCGTGAGTATTTGAGAGAGAGAAGGAGAATGCGA
                        W  E  S  G  K  R  E  Y  L  R  E  E  R
ATCAATCAAGCGACAATACTAACAACAGAGGGTGACGTCAAGATCTACAAGAAATAGACGAAT
R  R  M  R  I  N  Q  A  T  I  L  T  T  E
```

Figure S5 The coding regions for the precursor of the δ-subunit and for the ε-subunit of the F-ATPase in *Y. lipolytica*

Part A, the protein sequence of the δ-subunit is shown below the exons (red). Residues 1-24 are the mitochondrial import sequence of the protein and are removed during entry into the mitochondrion; part B, the ε-subunit. The sequence corresponds to nucleotides 92270-91792 in chromosome E of *Y. lipolytica* (NCBI database, accession number CR382131). The coding and non-coding nucleotide sequences are red and black, respectively. The sequence of the ε-subunit is shown below the exons. The mature protein lacks the N-terminal methionine residue.

A

```
MFRQSLRSIARTARTGTIGVRTYAEAVNPDVLKVSLVAPHQAIFTNKEVSQVNLPASSGE
MGVLANHVPTVEELAPGVVEVIESSGTASKYFVSGGFASILPGSKLSISTVEAHPLDAFS
SENIKSLLAEAQKNASSADETVAAEAAIEIEVLEALQAAVH
```

B

```
MSSVAKSTANKLDWTKIVSKLGLSGQTAAALTSFKKRNDEAKRILFELKQQPSNVDFAFY
KSTLKNTAIVDKIQSDVSKFTPSKANLSKQLNLIESFEAKALENAKETESVVLAELTDLE
KTLENIESARPFDQLTVDDVVKARPDVEEKVQDMVSKGRFEVPGYKEKFGDLVIM
```

C

```
MTNNYINSPLDQFIINNLLEINSPFLNLSTLNFSTFSLYTLFVVLVISLTFILSIGGESN
NLVKGSNWLIAIEAIFDTILNMVKGQIGGSVYGRYVPLVYTLFTFILVANLIGMVPYNFA
LSASLIYIIGISVSLWIGLTILGLFLNKAVFFSLFVPSGTPLPLVPVLVLIELLSYTARA
ISLGLRLAANTLSGHLLMSILGNLVKNLMSINYFTFIFGLIPLAGIFAIVILEFAIACIQ
AYVFAILTSSYLKDSIYLH
```

Figure S6 The amended sequences of the δ-, d- and a subunits of the F-ATPase from *P. angusta*

Parts A-C, the sequences of the δ-, d- and a-subunits, respectively, determined by re-sequencing segments of the genome of *P. angusta*. In comparison to sequences recorded in the NCBI database, the δ- and d-subunits each contains a single point substitution serine-alanine, and alanine-threonine, respectively, and the a subunit has two point substitutions, arginine-isoleucine and serine-asparagine. The positions of

the substitutions are shown in red. The mature δ- and a-subunits lack residues 1-23 and 1-7, respectively. Methionine-1 is removed post-translationally from subunit d, and the N-terminal serine residue of the mature protein is αN-acetylated.

Figure S7, part A

```
                    20                        40                        60
AGAYTLFGKAIPPHHLAIATIGTVVALVAPKPWSPKVKLEPKIDASSPEEEKFIKEYLEKHL

T1-3                                                        T6
T2-3
```

Figure S7, part B

```
                    20                        40                        60
AAPYVIFGAKVPPHWLAIGTILTVVGGIYGPKAFSSPAAAAPAATPAAAPSSEPELDVEKAINDFLASDKKE

T1            T3
T2            T4-5
T2-3
T3-5
```

Figure S7, part C

```
                    20                        40                        60
GAAYKVFGKTVQPHVLAISTFIATAAVASYFTTKPKTKNEGKNSSALSQQKSGESSNSDA

T1-2          T3-4          T8-9
                    80
MGKDDDVVKSIEGFLNDLEKDTRQDTKAN

T10
```

Figure S7 Characterisation of the sequences of subunits k and l from the F-ATPase of *P. angusta* and of subunit l from the F-ATPase of *S. cerevisiae*

In parts A, B and C, the tryptic peptides sequenced by tandem mass spectrometry are shown beneath the amino acid sequences of *P. angusta* subunits k and l and *S.*

cerevisiae subunit l, respectively. They represent 74, 98 and 79% of the complete protein sequences, respectively.

Table S5 Summary of peptide mass fingerprinting and tandem MS data for the F-ATPase from *S. cerevisiae*

	Peptide mass fingerprinting			Tandem MS	
Subunit[1]	MASCOT score	Peptides (n)	% coverage	Peptides (n)	Score
α	145/70	7	16	4	227/42
β	485/70	13	35	8	426/42
γ	394/70	12	41	4	327/42
δ	338/70	3	45	3	320/42
ε	615/70	6	91	5	542/42
OSCP	848/70	9	54	7	772/42
a	73/70	1	7	1	97/42
b	298/70	8	42	3	252/42
d	714/70	11	64	7	595/43
e	76/70	2	33	1	54/42
f	163/70	3	20	2	124/43
g	264/70	4	44	3	220/43
h	241/70	3	59	2	201/42
j	96/70	2	30	2	91/54
k	113/70	2	23	2	95/42
l	181/70	3	52	2	156/43

[1] In these experiments, no evidence was found for subunits c and ATP8.
